# Development and Validation of an RT-Droplet Digital PCR Assay for Detection of Peste des Petits Ruminants

**DOI:** 10.3390/vetsci13080841

**Published:** 2026-08-20

**Authors:** Rima Si, Jiao Xu, Xiaohua Wang, Yumeng Liu, Linlin Fang, Jiani Li, Yingli Wang, Jiarong Yu, Yi Zhai, Qinghua Wang, Jingyue Bao, Zhiliang Wang, Lin Yang

**Affiliations:** 1 Reference Laboratory for Peste des Petits Ruminants, China Animal Health and Epidemiology Center, Qingdao 266000, China; 2 College of Veterinary Medicine, Inner Mongolia Agricultural University, Hohhot 010010, China; 3 College of Veterinary Medicine, Qingdao Agricultural University, Qingdao 266105, China; 4 College of Veterinary Medicine, Nanjing Agricultural University, Nanjing 210095, China

**Keywords:** peste des petits ruminants virus, droplet digital PCR, clinical diagnosis, molucular detection

## Abstract

To achieve rapid, accurate, and quantitative detection of peste des petits ruminants virus (PPRV), we aligned the available PPRV genomic sequences, screened for conserved regions, and designed specific primers and probes accordingly. An RT-droplet digital PCR (RT-ddPCR) method was established, and a series of reaction conditions were optimized. Subsequently, the specificity, sensitivity, and repeatability of the method were subsequently evaluated. The results demonstrated that the RT-ddPCR method exhibited good specificity, with positive results obtained exclusively for PPRV and negative results for all other goat-derived viral or bacterial biological products and seed viruses tested. The method showed promising analytical sensitivity, with a LOD of 1.0 copies/μL for gene copy number. The method also demonstrated good repeatability, with coefficients of variation in all groups not exceeding 5%. In a preliminary clinical evaluation using 150 field samples, RT-ddPCR identified 113 positive cases, compared to 105 by RT-qPCR, with 8 additional low-copy-number positives detected exclusively by RT-ddPCR, and although the sample size is limited for precise diagnostic performance estimation, the assay showed 100% relative diagnostic sensitivity (105/105) and 82.2% relative diagnostic specificity (37/45), with almost perfect agreement between the two methods (κ = 0.87). As the positive signals in these discordant samples were too weak for independent confirmation by Sanger sequencing, they should be interpreted as “presumptive positives” requiring further investigation. The RT-ddPCR method established in this study enables sensitive and rapid detection of PPRV nucleic acid. However, detection of low-copy-number targets should be interpreted with caution, as it does not necessarily indicate active infection or epidemiologically significant virus circulation. This assay shows promise as a valuable research and surveillance tool for PPRV nucleic acid detection, particularly for confirming low-level viral RNA in samples near the detection limit of conventional methods, and could complement existing RT-qPCR-based approaches in PPR eradication programs.

## Simple Summary

We developed a reverse transcription droplet digital PCR (RT-ddPCR) method for the detection of all lineages of peste des petits ruminants virus (PPRV) and validated its performance characteristics. The assay demonstrated a limit of detection of 1.0 copies/μL for PPRV, showed no cross-reactivity with other common animal pathogens, and maintained good repeatability. Compared with RT-qPCR, it yielded consistent and highly accurate results for clinical samples. Therefore, this method shows promise as a complementary nucleic acid detection tool for peste des petits ruminants, particularly in research and surveillance applications.

## 1. Introduction

Peste des petits ruminants (PPR), also known as goat plague or ovine rinderpest, is a highly contagious transboundary viral disease affecting small ruminants, including sheep, goats, and wild ungulates [1,2]. The disease is caused by the peste des petits ruminants virus (PPRV), a member of the genus *Morbillivirus* (family *Paramyxoviridae*), with the current ICTV species designation *Morbillivirus caprinae* [3]. PPR is characterized by fever, erosive stomatitis, gastroenteritis, and pneumonia, with morbidity and mortality rates reaching up to 90% and 100%, respectively, in naive herds [1,4]. First identified in Côte d’Ivoire in 1942, PPR has since spread extensively across Africa and Asia (including China), and in recent years, Europe, posing a significant threat to food security and the livelihoods of small ruminant farmers in endemic regions [5,6,7,8]. The disease is currently listed as a notifiable disease by the World Organisation for Animal Health (WOAH), and global eradication efforts are ongoing under the PPR Global Control and Eradication Strategy (PPR-GCES) [9,10,11].

Currently, several diagnostic methods are available for the detection of PPRV, including virus isolation, serological assays, and molecular techniques [12,13]. Among these, reverse transcription quantitative polymerase chain reaction (RT-qPCR) has become the gold standard for laboratory diagnosis due to its high sensitivity, specificity, and rapid turnaround time [14]. The WOAH Terrestrial Manual recommends RT-qPCR as a confirmatory test for PPRV detection, targeting conserved regions of the N or F genes of the virus. However, RT-qPCR relies on standard curves for absolute quantification and is susceptible to PCR inhibitors, which are sometimes present in field samples [15,16]. Additionally, in the low-copy-number range, RT-qPCR often produces ambiguous Ct values, compromising the reliability of results for samples with low viral loads. These limitations highlight the need for alternative or complementary diagnostic approaches that can overcome these challenges [17].

Droplet digital PCR (ddPCR) is an emerging nucleic acid amplification technology that enables absolute quantification without the need for standard curves [18]. The principle of ddPCR is based on the partitioning of the reaction mixture into thousands of nanoliter-sized droplets, each serving as an individual reaction vessel. Following endpoint PCR amplification, droplets are read as positive or negative based on their fluorescence signal, and target concentration is calculated using Poisson statistics. Compared to RT-qPCR, ddPCR offers several distinct advantages: (1) absolute quantification without standard curves, eliminating the variability associated with standard curve preparation; (2) higher tolerance to PCR inhibitors due to the partitioning effect, making it particularly suitable for field samples with variable quality; (3) improved precision and reproducibility, especially at low target concentrations; and (4) high sensitivity for detecting rare targets [19,20,21,22]. These features make ddPCR an attractive tool for the detection of low-copy-number pathogens such as PPRV in clinical samples.

ddPCR has been increasingly applied for the detection and quantification of various animal pathogens, demonstrating significant advantages over traditional molecular diagnostic methods. This technology has been successfully applied across multiple animal species for different pathogens, showing remarkable sensitivity, specificity, and reproducibility in clinical settings. Yu et al. established a reverse transcription droplet digital PCR (RT-ddPCR) method for the detection of BVDV-1 and BTV in bovine semen, with analytical limits of detection (LOD) of 1.05 copies/μL and 0.662 copies/μL, respectively. The diagnostic detection rates of this method were higher than those of RT-qPCR [23]. Yu et al. developed the first triplex ddPCR method for the simultaneous detection of ASFV, PRV, and PPV, with limits of detection (LOD) of 0.08, 3.41, and 3.38 copies/μL, respectively, which were approximately 10-fold more sensitive than qPCR. Among 217 clinical samples, ddPCR detected 137 positive cases (63.1%), whereas qPCR detected only 115 positive cases (53.0%) [24]. Despite the advantages of ddPCR, its application for PPRV detection has been limited. To date, no study has systematically evaluated the ability of ddPCR to detect all four PPRV lineages (I–IV) in a single assay, nor has its performance compared with RT-qPCR been assessed using field samples with low viral loads. Given the genetic diversity of PPRV lineages circulating in different geographical regions [25], a pan-lineage detection method is essential for effective disease surveillance and eradication programs. Furthermore, the performance of ddPCR compared with RT-qPCR in detecting PPRV from field samples—particularly those with low viral loads—remains largely unexplored.

In this study, we aimed to develop and validate a highly sensitive (analytically), specific, and reproducible RT-ddPCR assay for the pan-lineage detection of PPRV targeting a highly conserved region of the nucleoprotein (N) gene. The assay was systematically optimized and evaluated for analytical sensitivity, specificity, reproducibility, and preliminary clinical performance using 150 field samples. We further compared the performance of the RT-ddPCR assay with that of RT-qPCR to assess its diagnostic utility. The results of this study suggest that the RT-ddPCR assay shows promise as a valuable tool for PPRV nucleic acid detection, particularly for identifying low-copy-number targets, and could complement existing RT-qPCR-based approaches in PPR eradication programs. However, further validation with larger, independently characterised specimen cohorts is needed to confirm its diagnostic accuracy and clinical utility.

## 2. Materials and Methods

### 2.1. Viruses and Plasmids

The plasmid standard containing the target gene fragment of PPRV was constructed and verified by Sangon Biotech (Shanghai) Co., Ltd. (Shanghai, China) based on the PPRV/XJYL/2013 (accession number: KM091959) strain sequence. The recombinant plasmid was propagated in Escherichia coli DH5α competent cells and purified using a Plasmid Mini Kit (QIAGEN, Hilden, Germany). Plasmid concentration was determined using a Qubit™ fluorometer (Thermo Fisher Scientific, Waltham, MA, USA), and the copy number was calculated based on the plasmid molecular weight. The viral strains used in this study, including PPRV lineages I to IV, goat pox virus (GPV), orf virus (ORFV), foot-and-mouth disease virus (FMDV), bluetongue virus 1 (BTV-1) and caprine herpesvirus 1 (CpHV-1) were stored at −80 °C in the National PPR Reference Laboratory (China Animal Health and Epidemiology Center, Qingdao, China). Viral nucleic acids were extracted from field samples using QIAamp^®^ Viral RNA Mini Kit (QIAGEN, Hilden, Germany) according to the manufacturer’s instructions and stored at −80 °C until use.

### 2.2. Primers and Probe

Primers and probe targeting a conserved region of the N gene of PPRV were designed to ensure broad-spectrum detection across all four lineages (I, II, III, and IV) of PPRV. 93 reference sequences of PPRV representing all lineages were retrieved from the GenBank database (Table S1). Multiple sequence alignment was performed using MEGA X software (10.1) to identify highly conserved regions suitable for primer and probe annealing. The primer-probe set was designed using Primer Express^®^ Software v3.0 (Thermo Fisher Scientific, Waltham, MA, USA) following the recommended guidelines for ddPCR assays. The criteria for primer and probe design were as follows: amplicon length between 70 and 150 bp; primer melting temperatures (Tm) between 58 °C and 62 °C with a ΔTm of ≤2 °C; probe Tm approximately 5–10 °C higher than the primers; GC content between 40% and 60%; and no secondary structures or primer-dimers, as predicted by the IDT OligoAnalyzer™ Tool (Integrated DNA Technologies, Coralville, IA, USA). The probe was labeled with a 5′ reporter dye (6-carboxyfluorescein, FAM) and a 3′ quencher (Black Hole Quencher 1, BHQ1). All oligonucleotides were synthesized by Sangon Biotech (Shanghai) Co., Ltd. and purified by high-performance liquid chromatography (HPLC). The sequences of the primers and probe are listed in Table 1.

### 2.3. RT-ddPCR Reaction Conditions

All RT-ddPCR assays were conducted using the Sniper DQ24 Pro™ ddPCR System (Suzhou, China), which integrates a droplet generator and automated fluorescence reader. The RT-ddPCR reaction was prepared in a total volume of 22 μL containing 11 μL of 2 × dPCR Probe Master Mix Plus (with Cy5.5) (RT003096A, Sniper, Suzhou, China), 1 μL each of forward and reverse primers at a concentration of 500 nM, 1 μL of the fluorescently labeled probe at a concentration of 450 nM, 2 μL of RNA template, and nuclease-free water to adjust to the final volume. The reaction mixture was thoroughly mixed and loaded onto the Sniper DQ24 Pro™ system for automated droplet generation and amplification according to the manufacturer’s instructions. The one-step RT-ddPCR reaction was performed using the Sniper DQ24 Pro™ ddPCR System with the following thermal cycling protocol: reverse transcription at 50 °C for 20 min; enzyme activation at 95 °C for 5 min; followed by 45 cycles of denaturation at 95 °C for 30 s and annealing/extension at 50 °C for 30 s. After amplification, the fluorescence signals were acquired through the FAM detection channel. Data were analyzed using the Sniper DQ24 Pro™ analysis software (1.0.1).

### 2.4. Analytical Sensitivity Evaluation

A preliminary analytical sensitivity screening was performed using a 10-fold serial dilution series of the plasmid standard ranging from 1 × 10^4^ to 1 × 10^−1^ copies/μL to determine the linear dynamic range and approximate detection threshold of the assay. Each dilution was tested in triplicate using the established RT-ddPCR system. Based on the preliminary results, the limit of detection (LOD) was further determined using 25 independent replicates at each of seven concentration levels (0.1, 0.25, 0.5, 0.75, 1.0, 1.25, and 1.5 copies/μL) to obtain a precise estimate of the lowest reliably detectable concentration. The LOD was defined as the lowest concentration achieving an observed positivity rate of ≥95%. Probit regression analysis was performed to model the concentration-response relationship using the equation: Probit (P) = a + b × log_10_(C). The 95% confidence intervals (CIs) for positivity rates were calculated using the Clopper-Pearson exact method.

### 2.5. Specificity Evaluation

To evaluate the analytical specificity of the RT-ddPCR assay, a panel of viral nucleic acids was tested, including PPRV lineages I, II, III, and IV, as well as clinically relevant viruses: GPV, ORFV, FMDV, BTV-1, CpHV-1 and *Pasteurella* spp. Viral nucleic acids were extracted and tested in triplicate using the established RT-ddPCR assay. A sample was considered positive when the fluorescence signal exceeded the threshold determined by the no-template control (NTC).

### 2.6. Repeatability Evaluation

To evaluate assay precision, intra-assay variability was determined by testing three replicates of each concentration (10^0^ to 10^4^ copies/μL) within a single run. Inter-assay variability was assessed by performing three independent runs on separate days using freshly prepared dilution series. The mean, standard deviation (SD), and coefficient of variation (CV%) were calculated for each concentration level.

### 2.7. Standard Curve of RT-ddPCR

To evaluate the linearity and accuracy of the ddPCR assay, a 10-fold serial dilution series of the plasmid standard was prepared to obtain seven concentration levels ranging from 1 × 10^0^ to 1 × 10^6^ copies/μL. Each dilution was tested in triplicate (three independent reaction wells) using the RT-ddPCR system. The expected concentrations were plotted against the measured concentrations (mean ± SD) after logarithmic transformation (log10). Linear regression analysis was performed to generate the regression equation and the coefficient of determination (R^2^).

### 2.8. Field Sample Detection

To evaluate the clinical performance of the RT-ddPCR assay in comparison with RT-qPCR, a total of 150 clinical samples were tested in parallel using both methods. RT-qPCR was performed as described previously [26] and served as the reference method. This RT-qPCR assay targets a conserved region of the PPRV P gene. As previously validated in our laboratory, this method has a limit of detection (LOD) of 4.8 copies/μL (based on plasmid DNA) and is capable of detecting PPRV lineages I–IV. To ensure a fair comparison between the two methods, all clinical samples were tested using the same RNA extracts. The diagnostic sensitivity, diagnostic specificity, and overall agreement between the two methods were calculated using standard diagnostic test evaluation formulas. Diagnostic sensitivity was defined as the proportion of RT-qPCR-positive samples that were correctly identified by RT-ddPCR, while specificity was defined as the proportion of RT-qPCR-negative samples that were correctly identified by RT-ddPCR. Overall agreement was calculated as the number of concordant results (both positive and both negative) divided by the total number of samples. All 95% confidence intervals (CIs) for proportions were calculated using the Wilson score method. Cohen’s kappa (κ) coefficient was used to assess the level of agreement beyond chance.

## 3. Results

### 3.1. Optimization of RT-ddPCR

**Figure 1:**
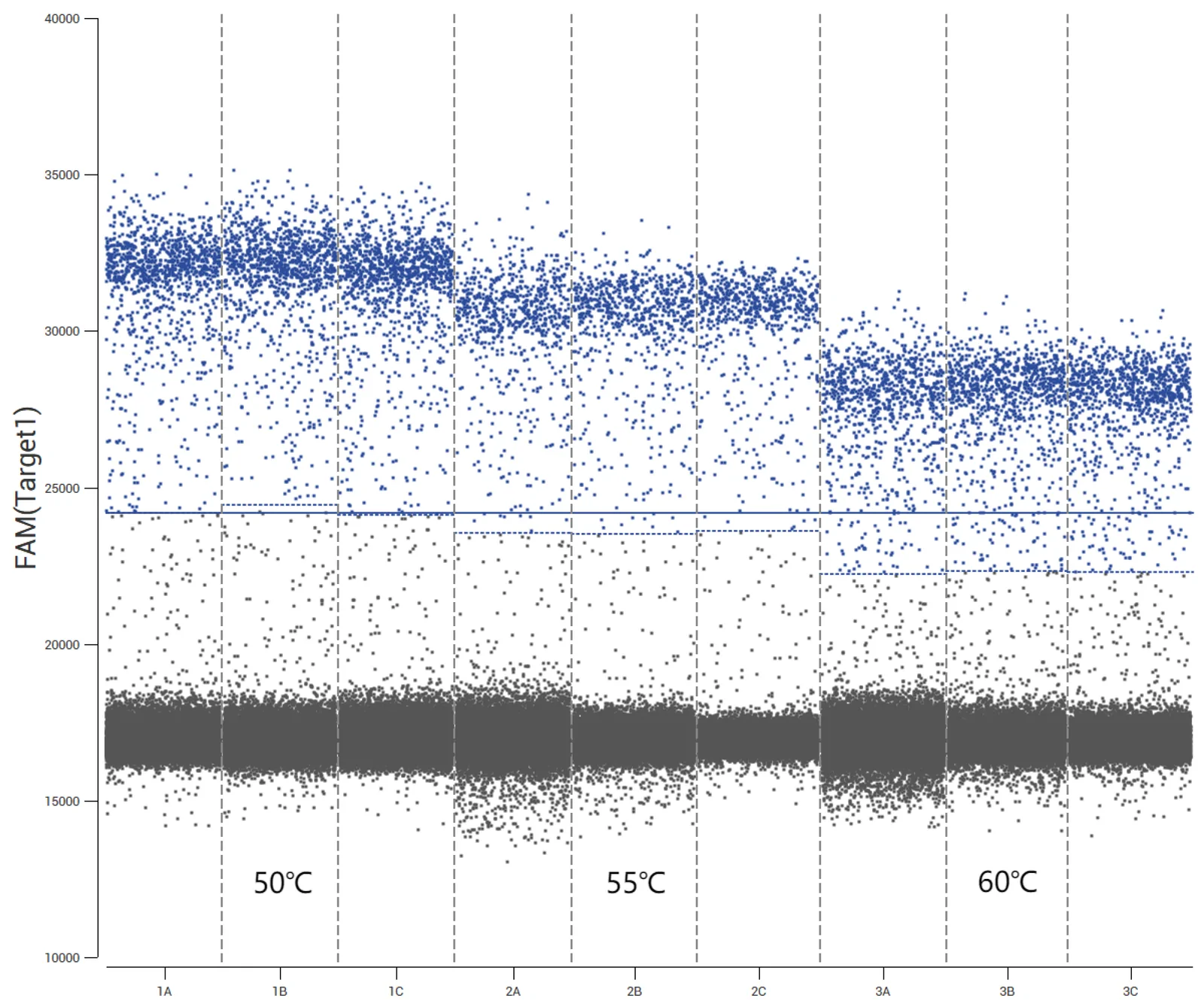
Optimization of the annealing temperature for the RT-ddPCR assay. The assay was performed at three different annealing temperatures (50 °C, 55 °C, and 60 °C) using the same template. The copy number readings (mean ± SD) from triplicate reactions are shown. The highest quantified copy numbers were consistently obtained at 50 °C.

**Figure 2:**
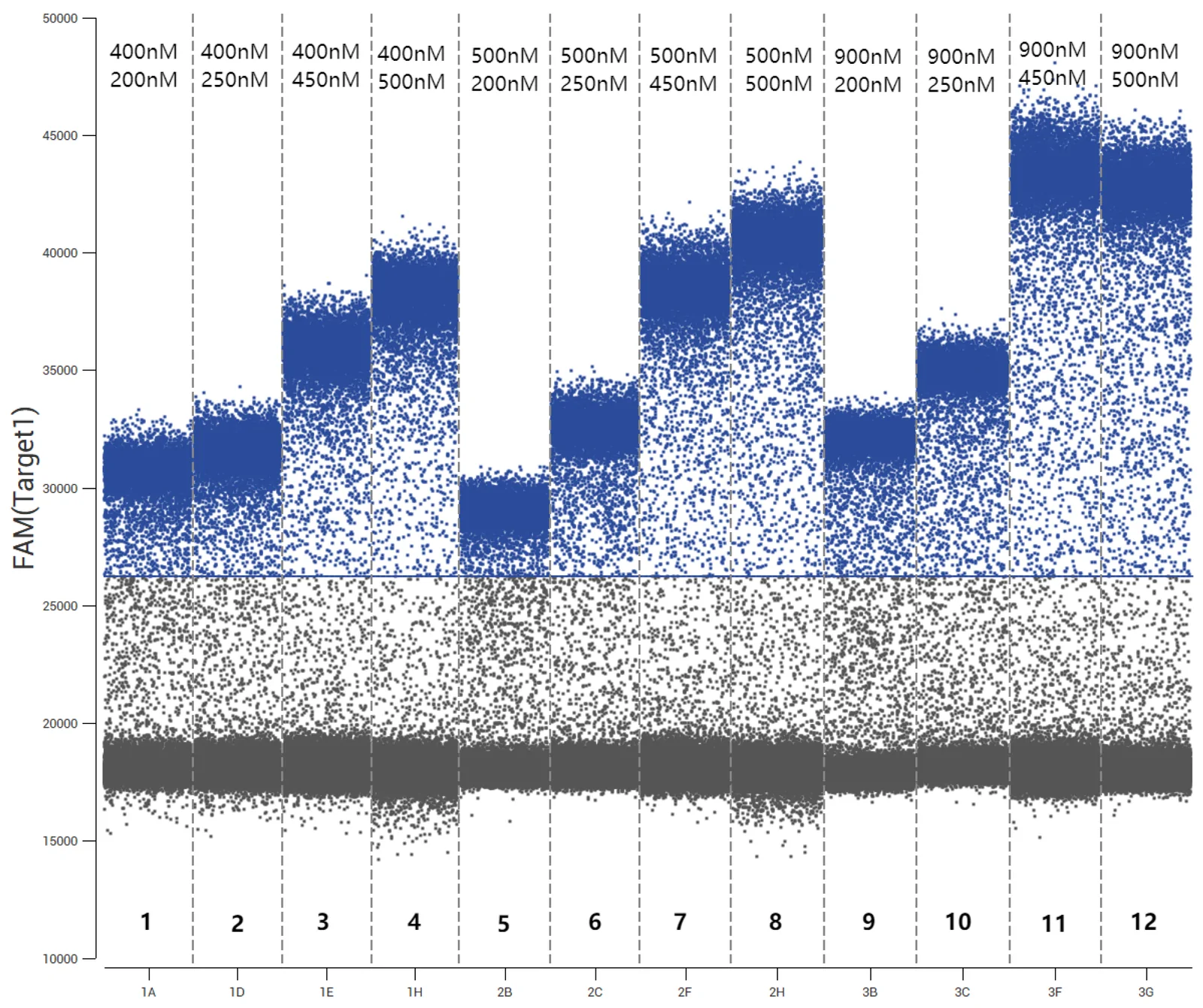
Optimization of primer and probe concentrations for the RT-ddPCR assay. Twelve different concentration combinations of primers and probe were evaluated in triplicate. The combination of 500 nM primers and 450 nM probe yielded the highest copy numbers.

To determine the optimal annealing temperature for the RT-ddPCR assay, three different annealing temperatures (50 °C, 55 °C, and 60 °C) were evaluated. At all three temperatures, clear discrimination between positive and negative droplets was observed, with high fluorescence intensity and good reproducibility across triplicate reactions. However, the highest copy number readings were consistently obtained at 50 °C (*p* < 0.05) (Figure 1). Considering the comprehensive performance-including droplet separation, fluorescence signal strength, reproducibility, and quantified copy number, 50 °C was selected as the optimal annealing temperature for all subsequent RT-ddPCR assays. To optimize the primer and probe concentrations for the RT-ddPCR assay, 12 different concentration combinations were evaluated, with each combination tested in triplicate. Among all combinations tested, the combination of 500 nM primers and 450 nM probe yielded the highest quantified copy numbers (*p* < 0.05). Additionally, this concentration combination exhibited the most distinct separation between positive and negative droplet clusters, optimal fluorescence signal intensity, and good reproducibility across three replicates. Based on these comprehensive criteria-including quantified copy number, droplet separation, fluorescence signal strength, and reproducibility-the concentration of 500 nM for primers and 450 nM for probe was selected as the optimal working concentration for all subsequent RT-ddPCR assays (Figure 2).

### 3.2. Analytical Sensitivity and Limit of Detection (LOD)

**Figure 3:**
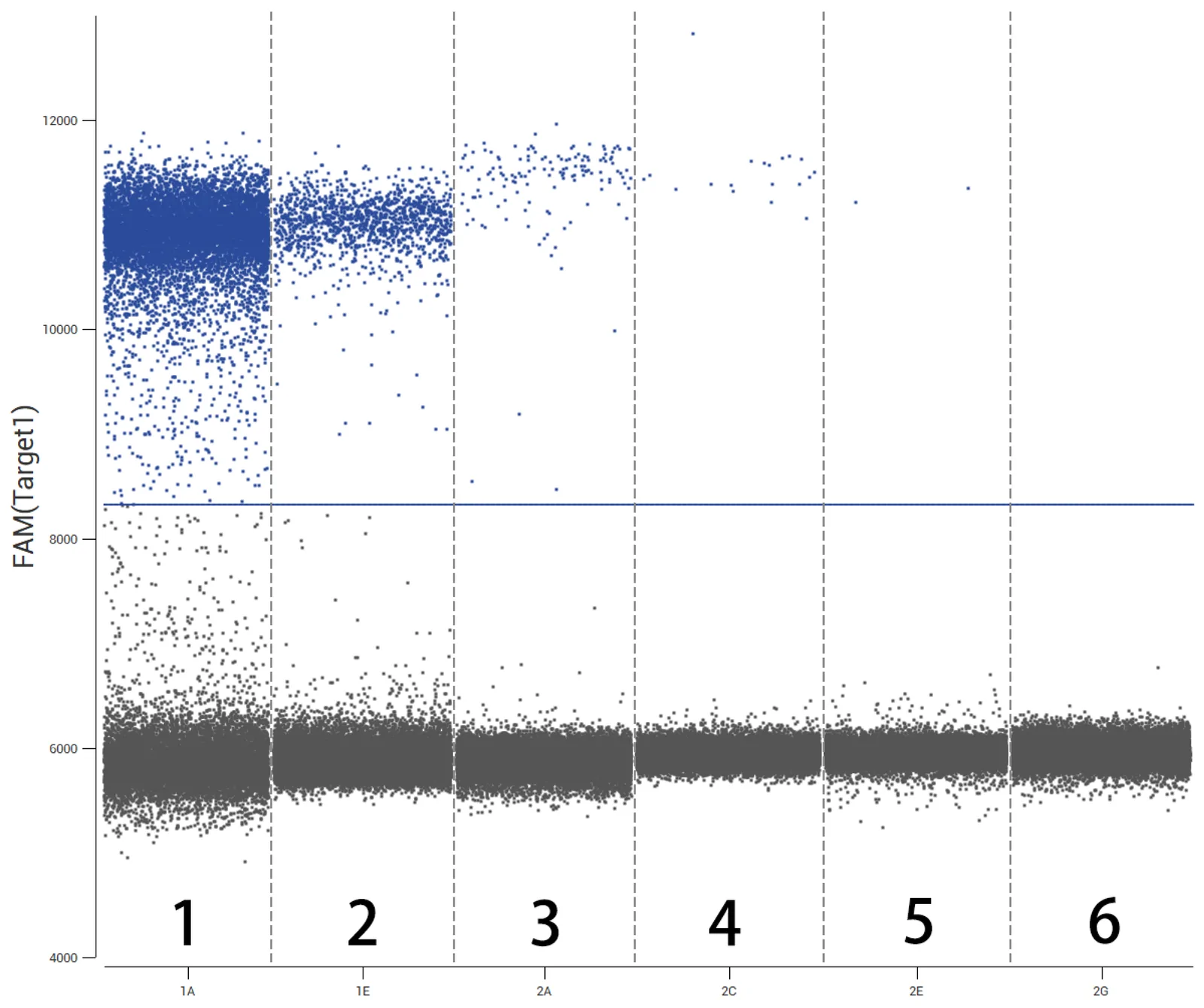
Analytical sensitivity of the RT-ddPCR assay. A 10-fold serial dilution of the plasmid standard ranging from 1 × 10^4^ to 1 × 10^−1^ copies/μL (1~6) was tested. Positive droplets were consistently detected at concentrations as low as 1 × 10^0^ copies/μL, demonstrating the high analytical sensitivity of the assay.

The results demonstrated that positive droplets were consistently detected at concentrations as low as 1 × 10^0^ copies/μL (Figure 3), indicating that the assay exhibited high analytical sensitivity with a detection capability approaching the single-copy level. The limit of detection (LOD) of the ddPCR assay was determined by testing 25 independent replicates at each concentration level across a dilution series ranging from 0.1 to 1.5 copies/μL. As summarized in Table 2, the positivity rates were 100% (25/25) at both 1.5 and 1.25 copies/μL, 96.0% (24/25) at 1.0 copies/μL, 64.0% (16/25) at 0.75 copies/μL, and 12.0% (3/25) at 0.5 copies/μL, with no positive signals detected at 0.25 copies/μL, 0.1 copies/μL, or in the no-template control. Probit regression analysis demonstrated a good fit (R^2^ = 0.993) with the equation: Probit(P) = 2.00 + 2.44 × log10(C). Based on the empirically defined criterion of ≥95% observed positivity, the functional LOD of the assay was established as 1.0 copies/μL (24/25 positive; 95% CI: 80.5–99.3%, Clopper-Pearson method). At the next lower tested concentration (0.75 copies/μL), the positivity rate dropped to 64.0%, confirming that this concentration is below the reliable detection threshold. These results demonstrate that the ddPCR assay can reliably detect the target at concentrations ≥ 1.0 copies/μL.

### 3.3. Specificity Evaluation

As shown in Table 3, positive droplet signals were exclusively observed in all PPRV lineage samples (lineages I, II, III, and IV), whereas no positive signals were detected for GPV, ORFV, FMDV, BTV-1, CpHV-1 or *Pasteurella* spp. The no-template controls (NTC) also remained negative across all runs. These results demonstrate that the RT-ddPCR assay exhibits high analytical specificity, enabling specific detection of all four PPRV lineages without cross-reactivity with other related viruses commonly found in small ruminants.

### 3.4. Repeatability Evaluation

The precision of the RT-ddPCR assay was evaluated by assessing both inter-assay and intra-assay variability across a 5-log dynamic range from 1 × 10^0^ to 1 × 10^4^ copies/μL. As summarized in Table 4, the inter-assay precision, determined from three independent runs, yielded coefficient of variation (CV) values ranging from 3.03% to 4.95% across all concentrations tested, with the lowest CV observed at 1 × 10^0^ copies/μL (3.03%) and the highest at 1 × 10^1^ copies/μL (4.95%). The intra-assay precision, assessed from three replicates within a single run, demonstrated even lower variability, with CV values ranging from 1.04% to 4.71%. Notably, at the lowest tested concentration (1 × 10^0^ copies/μL, equivalent to 1 copy/μL), the intra-assay CV was 2.97%, while the inter-assay CV was 3.03%, indicating excellent reproducibility even at the limit of quantification. Overall, all CV values were well below the commonly accepted threshold of 25%, demonstrating that the ddPCR assay exhibits robust and reproducible performance across the entire tested concentration range.

### 3.5. Standard Curve of RT-ddPCR

**Figure 4:**
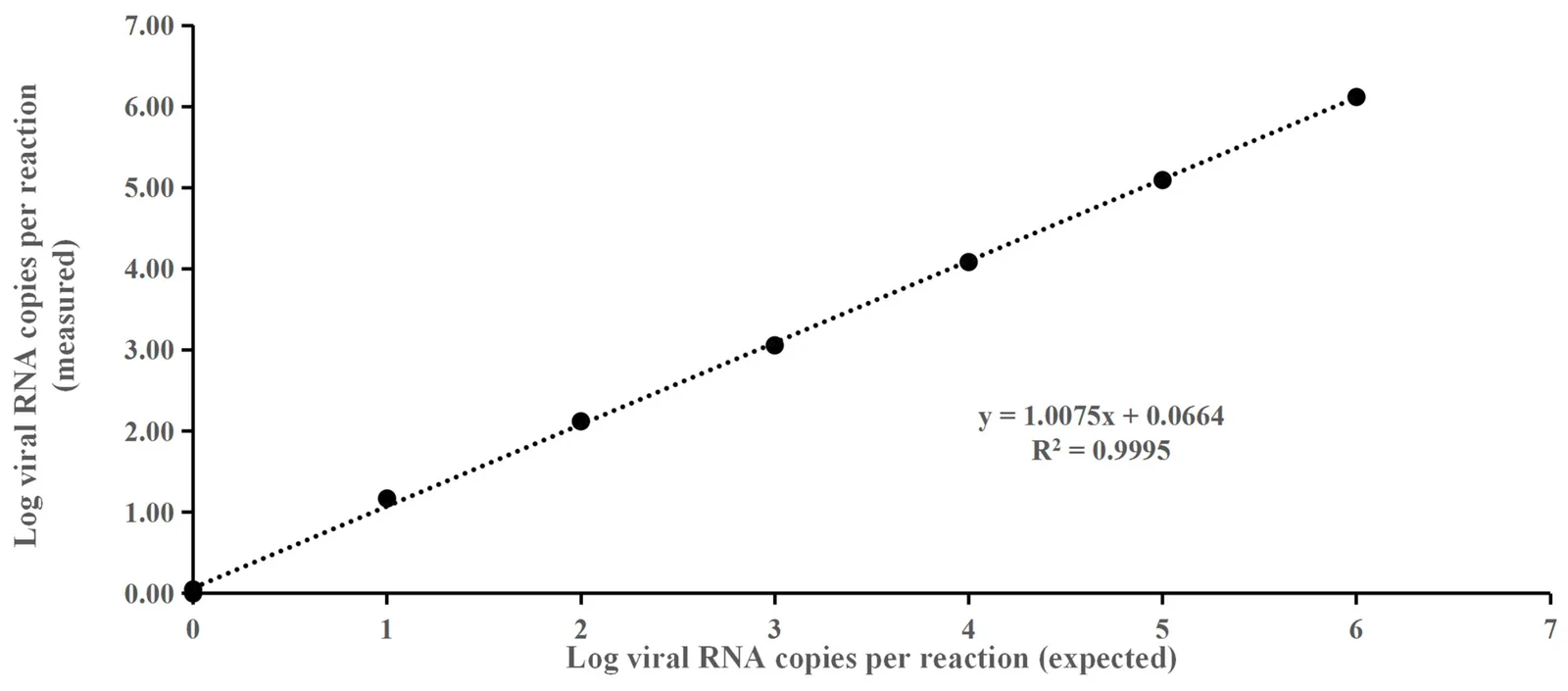
Standard curve of the RT-ddPCR assay. A 10-fold serial dilution series of the plasmid standard, ranging from 1 × 10^0^ to 1 × 10^6^ copies/μL, was tested in triplicate. The graph shows the linear regression analysis between the expected (log_10_) and measured (log_10_) concentrations. The regression equation was log_10_ (measured) = 1.0075 × log_10_ (expected) + 0.0664, with a coefficient of determination (R^2^ = 0.999), indicating excellent linearity across the entire dynamic range.

The linearity of the ddPCR assay was evaluated by testing a 10-fold dilution series of plasmid standards ranging from 1 × 10^0^ to 1 × 10^6^ copies/μL in triplicate. As shown in Table 5, a strong linear correlation was observed between the expected and measured concentrations across the entire dynamic range (Figure 4). Linear regression analysis yielded the equation: log_10_ (measured) = 1.0075 × log_10_ (expected) + 0.0664, with a coefficient of determination R^2^ = 0.999. This bias is consistent across all concentration levels and is likely attributable to the calibration of the plasmid standard or inherent instrument-specific variation. As the bias is uniform across the dynamic range, it does not compromise the relative quantification or the clinical concordance established in this study. The slope of 1.0075, which is close to the ideal value of 1.0, confirms the excellent proportional accuracy of the dilution series and the robustness of the ddPCR quantification across the tested range.

### 3.6. Clinical Performance and Diagnostic Sensitivity Evaluation

The clinical performance of the RT-ddPCR assay was evaluated using 150 field samples. The same RNA extracts from all samples were used for both RT-ddPCR and RT-qPCR to ensure a fair comparison. As shown in Table 6, ddPCR identified 113 positive samples, of which 105 were also positive by RT-qPCR, while 8 samples that were negative by RT-qPCR were positive by RT-ddPCR. All 37 samples that were negative by ddPCR were also negative by RT-qPCR, yielding no false-negative results for ddPCR relative to the reference method (detailed sample-by-sample results are provided in Supplementary Table S2). The relative diagnostic sensitivity of ddPCR was 100% (105/105; 95% CI: 96.6–100%), indicating that ddPCR did not miss any RT-qPCR-positive samples. The relative diagnostic specificity was 82.2% (37/45; 95% CI: 68.7–91.01%), reflecting the fact that ddPCR detected 8 additional positive cases that were negative by RT-qPCR. As the positive signals in these discordant samples were extremely weak (concentrations < 5 copies/μL), independent confirmation by Sanger sequencing could not be performed, they should be interpreted with caution, as their true infection status could not be confirmed without an independent reference method. Therefore, these results cannot be definitively classified as “true positives” and should be interpreted as “presumptive positives” requiring further investigation. The overall agreement between the two methods was 94.7% (142/150; 95% CI: 89.8–97.3%), with a Cohen’s kappa coefficient of 0.87 (95% CI: 0.78–0.95), indicating almost perfect agreement beyond chance. Due to the limited sample size, the confidence intervals for specificity are relatively wide, and these performance estimates should be considered preliminary. The discordant cases (ddPCR+/RT-qPCR-) were further analyzed and found to have ddPCR-measured concentrations ranging from 1.2 to 4.2 copies/μL, suggesting that these samples represented low-copy-number targets that were below the reliable detection limit of RT-qPCR but within the measurable range of RT-ddPCR. However, we acknowledge that these results require confirmation using an independent method (e.g., virus isolation or sequencing), and the current data should be interpreted as preliminary findings rather than definitive evidence of true infection.

## 4. Discussion

PPR remains one of the most economically devastating transboundary animal diseases (TADs) affecting small ruminant production systems worldwide. As one of the most destructive TADs, PPR places a severe socioeconomic burden on developing regions in particular; the disease is endemic across large swaths of Africa and Asia, where sheep and goats serve as critical livelihood assets for millions of smallholder farmers, pastoralists, and agropastoralists [27,28,29]. In these regions, PPR outbreaks result in direct losses through high morbidity and mortality as well as indirect losses from reduced milk and meat production, trade restrictions, and the substantial costs associated with vaccination and disease control measures. The socioeconomic impact extends beyond individual farm-level losses to affect national food security, rural livelihoods, and regional economic stability [30,31]. In response to this grave threat, the Food and Agriculture Organization (FAO) and WOAH launched the Global Eradication Programme (GEP) in 2015, building on the earlier PPR Global Control and Eradication Strategy, with the goal of eradicating the disease worldwide by 2030 [9,10,11]. The success of the GEP hinges on accurate, timely, and accessible diagnostic tools that can support surveillance, outbreak response, and certification of freedom from disease.

In recent years, researchers have developed a variety of novel techniques for PPRV detection to address the limitations of conventional methods in terms of sensitivity, convenience, and field applicability. In the field of molecular diagnostics, beyond the classic RT-PCR and real-time RT-qPCR, isothermal amplification technologies—including reverse transcription loop-mediated isothermal amplification (RT-LAMP) [32], reverse transcription recombinase polymerase amplification (RT-RPA) [33], and reverse transcription recombinase-aided amplification (RT-RAA) [34] have attracted considerable attention due to their independence from sophisticated temperature control equipment and operational simplicity. These methods are particularly well-suited for field rapid diagnosis in resource-limited settings. In the field of serological diagnostics, in addition to traditional virus neutralization tests (VNT) and ELISA, emerging technologies such as immunochromatographic lateral flow devices (LFD) [35] have also been applied for the rapid detection of PPRV antibodies.

In this study, we successfully developed and validated an RT-ddPCR assay for the detection of PPRV. The assay demonstrated good analytical sensitivity, specificity, reproducibility and performance compared to RT-qPCR, particularly for samples with low viral loads. To the best of our knowledge, this is the first RT-ddPCR method specifically designed for pan-lineage detection of all four PPRV lineages in a single reaction.

The high analytical sensitivity of RT-ddPCR (LOD of 1.0 copies/μL) holds profound implications for eradication programs, particularly in their later stages. As PPR prevalence progressively declines in regions approaching eradication, the ability to detect residual virus circulation becomes increasingly critical. In low-prevalence settings, conventional RT-qPCR may fail to detect samples with extremely low viral loads. This can lead to false-negative results, delayed detection of disease re-emergence, and ultimately, failure to certify disease-free status—a key milestone on the eradication pathway as assessed by the PPR Monitoring and Assessment Tool (PMAT). The RT-ddPCR assay established in this study, with an LOD of 1.0 copies/μL, demonstrates substantially higher analytical sensitivity than conventional RT-qPCR. For comparison, previously reported RT-qPCR methods targeting the N, F, or P genes of PPRV have demonstrated LODs ranging from 10 to 32 genomic copies/μL [36,37,38,39], representing a 10- to 32-fold higher detection limit than the RT-ddPCR assay developed in this study. The RT-ddPCR in this research can detect low-level infections that might be missed by conventional methods. The ability of this assay to detect and quantify PPRV at extremely low concentrations enhances early detection sensitivity, facilitating rapid intervention, limiting disease spread, and providing greater confidence in declaring disease-free status. However, it is critical to recognize that the detection of very small quantities of target nucleic acid does not necessarily indicate the presence of infectious virus, active infection, or epidemiologically relevant virus circulation. Nucleic acid may persist from non-infectious sources, including degraded viral RNA, vaccine residuals, or environmental contamination. Therefore, the increased analytical sensitivity of RT-ddPCR must be interpreted with caution.

In the clinical comparison performed in this study, all samples were tested using the same RNA extracts for both RT-ddPCR and RT-qPCR, thereby eliminating the potential confounding effect of RNA extraction efficiency on the comparative results and ensuring a fair and scientifically sound comparison between the two methods. The clinical performance of the RT-ddPCR assay was evaluated using 150 field samples, with RT-qPCR as the reference method. The 100% relative diagnostic sensitivity confirmed that RT-ddPCR did not miss any RT-qPCR-positive samples, while the 82.2% relative specificity reflected the detection of eight additional positive cases that were negative by RT-qPCR. Importantly, these discordant samples had RT-ddPCR-measured concentrations ranging from 1.2 to 4.2 copies/μL, indicating that they represented low-copy-number targets below the reliable detection limit of RT-qPCR. This observation is consistent with the known high analytical sensitivity of ddPCR in the low-copy-number range, as ddPCR is less susceptible to PCR inhibitors and does not rely on standard curve extrapolation for quantification [40,41]. The Cohen’s kappa coefficient of 0.87 (95% CI: 0.78–0.95) indicated almost perfect agreement between the two methods, further validating the clinical utility of the RT-ddPCR assay. It is important to note that the diagnostic sensitivity and specificity values reported in this study are relative to RT-qPCR as the reference method, not absolute measures of diagnostic accuracy. The eight discordant samples (ddPCR^+^/RT-qPCR^−^) could represent either true low-copy-number positives that were missed by RT-qPCR or false-positive results from ddPCR. Without confirmation using an independent method—such as virus isolation, sequencing, or an alternative nucleic acid detection assay—we cannot definitively determine their true infection status. Therefore, these results should be interpreted as preliminary evidence of the assay’s potential to detect low-copy-number targets.

Several limitations of this study should be acknowledged. First, the clinical sample size (n = 150) was relatively modest, and further validation with a larger cohort of field samples would strengthen the statistical power of the diagnostic performance evaluation. Second, the study focused on PPRV detection without evaluating the assay’s performance in differentiating vaccine strains from field isolates, which could be an important consideration for disease surveillance and control programs. Third, the analytical performance of the assay (including the LOD) was evaluated primarily using plasmid standards. As plasmid DNA is more stable and amplifies more efficiently than RNA, the LOD of 1.0 copies/μL determined with plasmid DNA may underestimate the true detection limit for clinical RNA samples. Furthermore, the performance of the assay with diverse sample matrices (e.g., blood, swabs, tissues) warrants further investigation. In addition, the relatively higher cost of RT-ddPCR compared to RT-qPCR represents a significant factor limiting its rapid replacement of the latter, and future research should also focus on reducing the cost of implementing this method.

Although this study evaluated 150 clinical samples, the number of RT-qPCR-negative samples (n = 45) was below the minimum of 62 negative samples required to estimate specificity with a precision of ±10% in a post hoc analysis. Therefore, the present findings should be considered preliminary, and larger multi-center validation studies with a greater number of negative samples are warranted to refine diagnostic performance estimates.

In conclusion, the RT-ddPCR assay developed in this study provides a promising analytically sensitive and specific tool for the detection of PPRV nucleic acid. Its ability to detect and quantify low-copy-number targets makes it a valuable complement to RT-qPCR, particularly for surveillance in the late stages of eradication programs, confirmatory testing of samples with ambiguous RT-qPCR results, and research applications requiring precise quantification. However, the detection of low-level RNA should be interpreted with caution and in conjunction with other diagnostic and epidemiological information, as nucleic acid detection does not necessarily indicate the presence of infectious virus or active infection. The assay should be considered as one component of a comprehensive diagnostic strategy rather than a standalone indicator of infection status. Future work should focus on evaluating the assay in larger field sample cohorts and exploring its utility in multiplex formats for simultaneous detection of multiple small ruminant pathogens.

## Figures and Tables

**Table 1:** Primer and Probe designed for this study.

Primers and Probe	Sequence (5′–3 ′)	Amplicon Size (bp)
Forward primer	TTCAGAACAAGTTTAGTGCAGG	108
Reverse primer	CCCCCCATTGAGTTCTCCA	
Probe	FAM–TGGAGCTATGCGATGGGTGTCGGAGT–BHQ1	/

**Table 2:** LOD Evaluation of RT-ddPCR.

Concentration (Copies/μL)	log_10_ (Concentration)	Positive Wells	Total Wells	Positivity Rate
1.5	0.176	25	25	100.00%
1.25	0.097	25	25	100.00%
1	0	24	25	96.00%
0.75	−0.125	16	25	64.00%
0.5	−0.301	3	25	12.00%
0.25	−0.602	0	25	0.00%
0.1	−1.000	0	25	0.00%
ddH_2_O	—	0	25	0.00%

**Table 3:** Specificity test of RT-ddPCR.

No.	Name of Strains	GenBank Accession Number	Lineage	Concentration (Copies/μL)		
1	China/XJYL/2013	KM091959	IV	3675	3121	3512
2	China/Tib/07	JF939201	IV	8371	8228	8301
3	China/JS/2014	MF443343	IV	6519	6901	6653
4	China/Tibet/Bharal/2008	JX217850	IV	7192	7301	7273
5	China/Tibet/2024	PP937138	IV	5381	5345	5383
6	PPRV/Sudan/Sinar/1972	OR286505	III	17,523	17,851	18,852
7	PPRV/Oman/1983	KJ867544	III	5373	5572	5498
8	Nigeria 75/1	HQ197753	II	11,632	11,021	12,851
9	PPRV/Ghana/NK1/2010	KJ466104	II	7210	7783	7631
10	PPRV/Cote_d Ivoire/1989	OL741724	I	19,824	20,121	20,652
11	PPRV/E32/1969	KP789375	I	14,323	14,797	14,738
12	GPV	/	/	Not detected	Not detected	Not detected
13	ORFV	/	/	Not detected	Not detected	Not detected
14	FMDV	/	/	Not detected	Not detected	Not detected
15	BTV-1	/	/	Not detected	Not detected	Not detected
16	CpHV-1	/	/	Not detected	Not detected	Not detected
17	Pasteurella	/	/	Not detected	Not detected	Not detected
18	H_2_O	/	/	Not detected	Not detected	Not detected

**Table 4:** Repeatability of RT-ddPCR.

**Inter-Assay Precision**						
**Plasmid Copy Number Copies/μL**				**Mean**	**SD**	**CV (%)**
1 × 10^4^	12,029.57	11,166.56	11,893.31	11,696.48	463.95	3.97%
1 × 10^3^	1298.47	1316.09	1390.86	1335.14	49.05	3.67%
1 × 10^2^	133.66	141.85	135.67	137.06	4.27	3.11%
1 × 10^1^	14.04	15.21	13.92	14.39	0.71	4.95%
1 × 10^0^	1.44	1.53	1.49	1.49	0.05	3.03%
**Intra-Assay Precision**						
**Plasmid Copy Number Copies/μL**				**Mean**	**SD**	**CV (%)**
1 × 10^4^	12,494.86	12,047.89	12,115.35	12,219.37	240.96	1.97%
1 × 10^3^	1221.31	1248.73	1189.06	1219.70	29.87	2.45%
1 × 10^2^	138.52	135.92	138.28	137.57	1.44	1.04%
1 × 10^1^	12.38	13.26	12.13	12.59	0.59	4.71%
1 × 10^0^	0.98	1.04	1.01	1.01	0.03	2.97%

**Table 5:** Standard curve of RT-ddPCR.

Expected Concentration (Copies/μL)	log_10_ (Expected)	Measured Concentration (Mean ± SD, Copies/μL)	Log_10_ (Measured)
1 × 10^6^	6	1.31 × 10^6^ ± 2.1 × 10^4^	6.117
1 × 10^5^	5	1.24 × 10^5^ ± 3.5 × 10^3^	5.093
1 × 10^4^	4	1.21 × 10^4^ ± 4.7 × 10^2^	4.083
1 × 10^3^	3	1.14 × 10^3^ ± 3.1 × 10^1^	3.057
1 × 10^2^	2	1.32 × 10^2^ ± 3.81	2.121
1 × 10^1^	1	1.48 × 10^1^ ± 0.72	1.170
1 × 10^0^	0	1.12 ± 0.04	0.049

**Table 6:** Evaluation of diagnostic accuracy.

		RT-qPCR (Q)		
		Positive	Negative	Total
ddPCR (D)	Positive	105	8	113
	Negative	0	37	37
	Total	105	45	150
Parameter		Value (D/Q)	95% CI	
Relative diagnostic sensitivity		100% (105/105)	96.6–100%	
Relative diagnostic specificity		82.2% (37/45)	68.7–91.1%	
Overall agreement		94.7% (142/150)	89.8–97.3%	
Cohen’s kappa		0.87	0.78–0.95	

## Data Availability

The original contributions presented in this study are included in the article. Further inquiries can be directed to the corresponding author.

## Supplementary Materials

The following supporting information can be downloaded at: https://www.mdpi.com/article/10.3390/vetsci13080841/s1, Table S1: Details of reference strains used in this study; Table S2: Information of clinical samples and detective results of RT-qPCR and RT-ddPCR.
