# Supplementary material for: Development and Validation of an RT-Droplet Digital PCR Assay for Detection of Peste des Petits Ruminants
Source: Vet Sci. 2026 Aug 20;13(8):841. doi: 10.3390/vetsci13080841 (PMC13517568; doi:10.3390/vetsci13080841)
Supplement: Supplementary file 1 [file vetsci-13-00841-s001.zip › vetsci-4488577-supplementary.pdf]

Table S1 Details of reference strains used in this study

Strain	Collection Date	Country	Host	GenBank Acc. No.	Reference (PubMed ID)	Lineage
ICV89	1989	Cote d'Ivoire	Capra hircus	EU267273	18541325	I
E32/1969	1969.9.3	Senegal	Capra hircus	KP789375	25953180	I
PPRV/Senegal/Dakar/1994	1994	Senegal	Capra hircus	OR286474	38476867	I
PPRV/BurkinaFaso/Ouagadoudou/1988	1988	Burkina Faso	Capra hircus	OR286475	38476867	I
Nigeria 76/1	1976	Nigeria	Capra hircus	EU267274	18541325	II
CIV 01P 2009	2009	Cote d'Ivoire	Capra hircus	KR781451	/	II
Ghana NK1 2010	2010	Ghana	Capra hircus	KJ466104	25150987	II
Nigeria/75/1	1975	Nigeria	Capra hircus	HQ197753	/	II
SnDk11I13	2013.3.11	Senegal	Capra hircus	KM212177	25291758	II
Benin/10/2011	2011.5.21	Benin	Ovis aries	KR781449	26801518	II
Benin/B1/1969	1969	Benin	Capra hircus	KR781450	26801518	II
NGKW2012-MSLN	2012.5.9	Nigeria	Capra hircus	KR828814	/	II
Lib/2015	2015.7.8	Liberia	Capra hircus	KU236379	/	II
PPRV/Sierra Leone/048/2011	2011.12.17	Sierra Leone	Capra hircus	MF741712	/	II
PPRV/Senegal/Nguekhokh/2/2010	2010	Senegal	Capra hircus	OR286476	38476867	II
PPRV/Mauritania/Tarza/2012	2012	Mauritania	Ovis aries	OR286478	38476867	II
PPRV/Guinea/Dalaba/2013	2013	Guinea	Capra hircus	OR286479	38476867	II
PPRV/Mali/Kolondieba/4/2013	2013.7.27	Mali	Capra hircus	OR286480	38476867	II
PPRV/Senegal/Pakour/2/2013	2013.3.8	Senegal	Capra hircus	OR286483	38476867	II
PPRV/Mali/Sagabari/10/2014	2014.7.10	Mali	Capra hircus	OR286484	38476867	II
PPRV/Senegal/SakhMecke/3/2012	2012.5.21	Senegal	Capra hircus	OR286485	38476867	II
PPRV/Senegal/Soum/2/2012	2012.3.8	Senegal	Capra hircus	OR286488	38476867	II
PPRV/Senegal/Ngairing/1/2010	2010.4.28	Senegal	Capra hircus	OR286489	38476867	II
PPRV/Mali/Segou/3/2014	2014	Mali	Capra hircus	OR286490	38476867	II

PPRV/Mali/Kolondieba/1/2013	2013.7.27	Mali	Capra hircus	OR286497	38476867	II
PPRV/Ghana/AttaBagbe/2014	2014.3.3	Ghana	Capra hircus	OR286498	38476867	II
PPRV/Senegal/Kedougou/31/2016	2016	Senegal	Capra hircus	OR286499	38476867	II
PPRV/Mali/Bamako/1999	1999	Mali	Capra hircus	OR286500	38476867	II
PPRV/Ghana/Accra/1978	1986.6.19	Ghana	Capra hircus	OR286502	38476867	II
PPRV/Mali/Kayes/39b/2016	2016.4.6	Mali	Capra hircus	OR286503	38476867	II
Ethiopia 1994	1994	Ethiopia	Capra hircus	KJ867540	25342675	III
Oman 1983	1983	Oman	Capra hircus	KJ867544	25342675	III
UAE 1986	1986	UAE	Capra hircus	KJ867545	25342675	III
Uganda 2012	2012	Uganda	Capra hircus	KJ867543	25342675	III
KN5/2011	2011.5	Kenya	Capra hircus	KM463083	25342678	III
Tanzania/2016/Ngorongoro	2016	Tanzania	Capra hircus	MW960272	/	III
Tanzania_Momba_2018	2018	Tanzania	Capra hircus	MZ322753	/	III
PPRV/Sudan/Sinar/1972	1972	Sudan	Capra hircus	OR286505	38476867	III
China/Tibet/30/2007	2007.8	China	Capra hircus	FJ905304	20813134	IV
China/33/2007	2007	China	Capra hircus	KX421388	28734191	IV
China/Tibet/Bharal/2008	2008	China	Pseudois nayaur	JX217850	22966182	IV
Turkey 2000	2000	Turkey	Capra hircus	NC006383	15845262	IV
Ethiopia 2010	2010	Ethiopia	Capra hircus	KJ867541	/	IV
India TN Gingee 2014	2014.9.16	India	Capra hircus	KR261605	26358594	IV
Morocco 2008	2008	Morocco	Capra hircus	KC594074	23661470	IV
Turkey 2000	2000	Turkey	Ovis aries	AJ849636	15845262	IV
China/Tib/07	2007.12	China	Capra hircus	JF939201	/	IV
China/XJYL/2013	2013.11.30	China	Capra hircus	KM091959	25301639	IV
China/BJ/2014	2014.8.16	China	Capra hircus	KP260624	25676751	IV
Izatnagar/94	1994	India	Capra hircus	KR140086	/	IV
India/TN/Gingee/2014	2014.9.16	India	Capra hircus	KR261605	26358594	IV
CH/GDDG/2014	2014.12.5	China	Capra hircus	KP868655	26573282	IV
IND/TN/VM/2014/02	2014.11.23	India	Capra hircus	KT860063	/	IV

Sungri/96	1996	India	Capra hircus	KF727981	24526640	IV
PPRV/Mongolia/9/2016	2016.9	Mongolia	Capra hircus Ovis aries	KY888168	28667443	IV
S15	2015.11.11	Algeria	Capra hircus	KY885100	28426782	IV
China/XJ2/2013	2013.12.20	China	Capra hircus	KX421384	28734191	IV
China/XJ3/2013	2013.12.21	China	Ovis aries	KX421385	28734191	IV
China/XJ4/2013	2013.12.22	China	Capra hircus	KX421386	28734191	IV
China/XJ5/2013	2013.12.29	China	Capra hircus	KX421387	28734191	IV
ChinaZJ2014	2014.4.25	China	Capra hircus	MF443335	28734191	IV
ChinaYN2014	2014.4.1	China	Capra hircus	MF443336	28734191	IV
ChinaSX2014	2014.4.5	China	Capra hircus	MF443337	28734191	IV
ChinaSC2014	2014.6.10	China	Capra hircus	MF443338	28734191	IV
ChinaSaX2014	2014.4.1	China	Capra hircus	MF443339	28734191	IV
ChinaNX2014	2014.2.17	China	Ovis aries	MF443340	28734191	IV
ChinaLN2014	2014.3.17	China	Capra hircus	MF443341	28734191	IV
ChinaJX2014	2014.4.1	China	Capra hircus	MF443342	28734191	IV
ChinaJS2014	2014.4.2	China	Capra hircus	MF443343	28734191	IV
ChinaJL2014	2014.4.1	China	Ovis aries	MF443344	28734191	IV
ChinaHN2014	2014.4.25	China	Capra hircus	MF443345	28734191	IV
ChinaHLJ2014	2014.3.31	China	Capra hircus	MF443346	28734191	IV
ChinaHeN2014	2014.4.3	China	Capra hircus	MF443347	28734191	IV
ChinaHB2014	2014.4.3	China	Capra hircus	MF443348	28734191	IV
Georgia/Tbilisi/2016	2016.1.14	Georgia	Capra hircus	MF737202	/	IV
PPRV/Bangladesh/BD2/2008	2008.5	Bangladesh	Capra hircus	MG581412	/	IV
Turkey/Central_Anatolia/2018	2018.9	Turkey	Ovis aries	MN657232	/	IV
Kurdistan/2011	2011.2	Iraq	Capra aegagrus	MK408669	/	IV
PPRV/saiga3/Mongolia/2017-01	2017.1	Mongolia	Saiga tatarica	MZ061719	34754511	IV
PPRV/saiga4/Mongolia/2017-01	2017.1	Mongolia	Saiga tatarica	MZ061720	34754511	IV
PPRV/Siberian_ibex/Mongolia/2017-01	2017.1	Mongolia	Capra sibirica	MZ061721	34754511	IV
PPRV/Goitered_gazelle/Mongolia/2017-01	2017.1	Mongolia	Gazella subgutturosa	MZ061722	34754511	IV
PPRV/DRC/Tshela/27/2012	2012.3	Democratic Republic of the Congo	Capra hircus	OL310685	/	IV
PPRV/PPRV/Isra	1997	Israel	Capra hircus	OL310687	28545149	IV

el-2536/Hebron/1997						
PPRV/Israel-4522/Tzora/1998	1998	Israel	Capra hircus	OL310688	28545149	IV
PPRV/Israel-2233/Beir-El-Makhsour/2003	2003	Israel	Capra hircus	OL310694	28545149	IV
PPRV/Israel-1277/Jordan-Valley/2004	2004	Israel	Capra hircus	OL310695	28545149	IV
PPRV/Bangladesh/BD12/2015	2015	Bangladesh	Capra hircus	OK274213	34848354	IV
PPRV/Bangladesh/BD17/2017	2017	Bangladesh	Capra hircus	OK274214	34848354	IV
PPRV/Ethiopia/Habru/2014	2014	Ethiopia	Capra hircus	ON110960	/	IV
China-HLJ/01/2013(c2)	2013	China	Capra hircus	OP066374	/	IV
PPRV/India/Calcutta/1995	1995.3.22	India	Capra hircus	OR286504	38476867	IV

Table S2. Information of clinical samples and detective results of RT-qPCR and RT-ddPCR.

Sample ID	Sample Type	RT-qPCR (Ct value)	Lineage	RT-ddPCR (copies/μL)
1	Lymph node	19.35	IV	3.5×10^{6}
2	Swab	24.65	II	4.5×10^{4}
3	Lung	33.01	IV	4.2×10^{1}
4	WOAH*	28.14	III	1.5×10^{3}
5	Swab	31.89	IV	8.9×10^{1}
6	WOAH	19.21	I	3.3×10^{6}
7	Bronchus	/	–	/
8	Lung	32.16	IV	1.5×10^{2}
9	Spleen	25.96	IV	7.1×10^{4}
10	Lymph node	21.98	IV	5.6×10^{5}
11	WOAH	30.95	I	2.2×10^{2}
12	Spleen	26.74	IV	9.8×10^{3}
13	Spleen	/	–	/
14	Lymph node	/	–	/
15	Spleen	/	–	/
16	Bronchus	19.83	IV	3.4×10^{6}
17	WOAH	28.56	III	3.6×10^{3}
18	WOAH	28.88	IV	3.5×10^{3}
19	Swab	34.33	Unknown	5.7×10^{1}

20	Lymph node	/	Unknown	2.8×10^0
21	WOAH	/	−	/
22	Lymph node	18.21	IV	4.8×10^6
23	Swab	18.97	IV	4.8×10^6
24	WOAH	27.18	III	2.3×10^3
25	Bronchus	19.63	II	3.7×10^6
26	Bronchus	/	−	/
27	Lung	25.71	IV	4.3×10^4
28	Swab	31.14	IV	1.7×10^2
29	WOAH	30.89	II	2.1×10^2
30	Swab	30.82	IV	2.1×10^2
31	Lymph node	31.67	IV	1.7×10^2
32	Spleen	18.85	IV	4.2×10^6
33	Spleen	/	−	/
34	WOAH	/	−	/
35	Lung	25.75	IV	4.1×10^4
36	Bronchus	/	Unknown	3.2×10^0
37	Lymph node	28.91	IV	8.6×10^3
38	Lymph node	/	Unknown	4.2×10^0
39	WOAH	28.88	I	3.4×10^3
40	WOAH	29.15	I	2.6×10^2
41	WOAH	30.38	III	1.3×10^2
42	Lymph node	/	−	/
43	Bronchus	28.74	III	4.1×10^3
44	Swab	/	−	/
45	WOAH	/	−	/
46	Spleen	/	−	/
47	Lymph node	/	−	/
48	Bronchus	/	−	/
49	WOAH	34.05	Unknown	6.2×10^1
50	WOAH	18.74	II	5.1×10^6
51	WOAH	17.98	I	9.7×10^6
52	Swab	26.51	II	3.7×10^4
53	Spleen	/	−	/
54	Lymph node	26.66	II	3.7×10^4
55	WOAH	28.31	III	2.9×10^4
56	Lymph node	27.98	II	3.3×10^4
57	Lymph node	/	−	/
58	Lymph node	/	−	/
59	Spleen	/	Unkno	1.7×10^0

			wn	
60	Lymph node	/	−	/
61	Bronchus	/	−	/
62	Bronchus	/	Unknown	1.2×10^0
63	Lymph node	22.45	IV	8.2×10^5
64	Swab	26.78	II	5.6×10^4
65	Lung	30.12	IV	3.8×10^2
66	WOAH	19.87	I	4.1×10^6
67	WOAH	24.33	III	2.3×10^5
68	Bronchus	33.56	IV	6.7×10^1
69	Lymph node	/	−	/
70	Swab	29.45	II	1.1×10^3
71	Lung	27.89	IV	3.2×10^4
72	WOAH	21.56	III	9.8×10^5
73	Spleen	31.23	IV	2.5×10^2
74	Bronchus	/	−	/
75	WOAH	18.92	I	6.7×10^6
76	Swab	25.67	IV	8.9×10^4
77	WOAH	32.78	II	8.5×10^1
78	WOAH	23.45	III	3.4×10^5
79	Spleen	28.12	IV	5.6×10^3
80	WOAH	30.89	I	3.3×10^2
81	Lymph node	31.72	IV	3.1×10^2
82	Swab	24.56	IV	8.8×10^5
83	WOAH	/	−	/
84	WOAH	19.34	II	5.5×10^6
85	Spleen	26.45	IV	7.8×10^4
86	WOAH	31.78	III	1.9×10^2
87	Lymph node	22.78	IV	4.5×10^5
88	Swab	/	−	/
89	WOAH	28.56	I	4.2×10^3
90	Lung	/	Unknown	2.9×10^0
91	Spleen	/	−	/
92	WOAH	27.34	II	2.6×10^4
93	Lymph node	30.67	IV	1.8×10^2
94	WOAH	24.12	III	1.2×10^5
95	WOAH	/	−	/
96	WOAH	29.78	I	7.5×10^3
97	Spleen	32.45	IV	3.4×10^2
98	Bronchus	20.56	II	2.8×10^6
99	Lymph node	/	Unknown	4.1×10^0

100	Swab	/	−	/
101	Lung	26.89	IV	5.6×10^4
102	Lung	31.45	III	2.3×10^2
103	WOAH	23.78	I	3.7×10^5
104	Bronchus	/	−	/
105	Lymph node	28.34	II	6.8×10^3
106	Swab	33.12	IV	7.2×10^1
107	WOAH	19.56	III	5.4×10^6
108	Lung	/	Unknown	3.1×10^0
109	Spleen	30.56	I	4.5×10^2
110	Bronchus	24.89	II	1.5×10^5
111	Lymph node	/	−	/
112	Swab	29.12	IV	8.7×10^3
113	Swab	32.67	IV	6.5×10^1
114	WOAH	22.34	III	6.7×10^5
115	Spleen	/	−	/
116	Bronchus	26.78	IV	3.2×10^4
117	Lymph node	31.89	II	2.6×10^2
118	WOAH	18.45	I	8.9×10^6
119	Swab	28.78	IV	4.5×10^3
120	Lung	/	−	/
121	WOAH	24.56	III	1.8×10^5
122	Bronchus	30.23	IV	3.7×10^2
123	Lymph node	33.78	IV	5.8×10^1
124	Swab	/	−	/
125	WOAH	27.12	II	9.8×10^3
126	WOAH	29.89	I	3.4×10^3
127	Spleen	/	−	/
128	Bronchus	21.67	IV	6.5×10^5
129	WOAH	32.34	III	1.2×10^2
130	Swab	25.89	IV	7.6×10^4
131	Spleen	/	−	/
132	Lung	30.78	II	2.8×10^2
133	WOAH	28.56	I	5.6×10^3
134	Bronchus	/	−	/
135	Lymph node	23.12	IV	4.2×10^5
136	Swab	34.78	IV	4.9×10^1
137	WOAH	20.45	II	3.2×10^6
138	WOAH	27.56	III	5.7×10^4
139	Spleen	31.12	IV	3.9×10^2
140	WOAH	24.78	I	1.3×10^5
141	Lymph node	/	−	/
142	Swab	29.56	IV	7.2×10^3

143	WOAH	33.45	II	7.8×10^1
144	WOAH	22.89	III	5.4×10^5
145	Spleen	/	−	/
146	Bronchus	26.34	II	4.8×10^4
147	Lymph node	31.56	IV	1.5×10^2
148	WOAH	19.78	I	7.8×10^6
149	Swab	28.45	IV	5.6×10^3
150	Lung	/	−	/

*WOAH: Viral nucleic acid obtained from WOAH.
